# Human Papillomavirus T-Cell Cross-reactivity in Cervical Cancer: Implications for Immunotherapy Clinical Trial Design

**DOI:** 10.1001/jamanetworkopen.2018.0706

**Published:** 2018-07-06

**Authors:** Sarah R. Helman, Sanja Stevanović, Tracy E. Campbell, Mei Li M. Kwong, Stacey L. Doran, William C. Faquin, Christian S. Hinrichs

**Affiliations:** 1 Experimental Transplantation and Immunology Branch, National Cancer Institute, Bethesda, Maryland; 2 School of Medicine, University of Pittsburgh, Pittsburgh, Pennsylvania; 3 Department of Surgery, MedStar Washington Hospital Center, Washington, DC; 4 Department of Pathology, Massachusetts General Hospital, Boston

## Abstract

**Importance:**

Clinical trials are testing vaccines that target human papillomavirus 16 (HPV-16) oncoproteins for the treatment of cervical cancer regardless of the HPV type of the tumor. For patients with HPV-18–positive cancers, this strategy relies on cross-reactivity of HPV-16–reactive T cells against the HPV-18 oncoproteins.

**Objectives:**

To determine the prevalence of HPV-16 and HPV-18 metastatic cervical cancers in women enrolling in clinical trials at a US medical center and to assess whether HPV oncoprotein–targeting tumor-infiltrating lymphocytes (TILs) and T-cell receptors (TCRs) possess HPV-16/HPV-18 oncoprotein cross-reactivity.

**Design, Setting, and Participants:**

This study was conducted at the National Institutes of Health Clinical Center, a tertiary care research hospital in the United States. The HPV type of the tumors from 65 consecutive patients with cervical cancer who were evaluated for participation in clinical trials was determined by retrospective medical record review. Immunological assays testing HPV cross-reactivity were conducted on all available archived samples of oncoprotein-reactive TILs from HPV-positive tumors (n = 16) and on a library of previously identified TCRs (n = 10).

**Interventions:**

The HPV genotype of each patient’s tumor was determined. The cross-reactivity of archived TILs and a library of TCRs was assessed.

**Main Outcomes and Measures:**

The main outcomes were the prevalence of each HPV genotype and the frequency of TILs or TCRs with HPV oncoprotein–T-cell cross-reactivity. Cross-reactivity was assessed by enzyme-linked immunospot assays and interferon-γ production assays.

**Results:**

The median (range) age of 65 referred patients was 44 (24-64) years. Ethnicity was recorded for 39 of 65 patients; 35 (89.7%) were white, 3 (7.7%) were Asian, and 1 (2.6%) was American Indian/Alaskan Native. Histologic tumor subtype was recorded for 41 of 65 patients; 25 (61.0%) were squamous cell carcinomas, 12 (29.3%) were adenocarcinomas, 2 (4.9%) were adenosquamous cell carcinomas, and 2 (4.9%) were neuroendocrine tumors. Thirty-nine of 65 patients (60.0%) had HPV-16–positive tumors and 21 patients (32.3%) had HPV-18–positive tumors. In the analysis of cross-reactivity, 1 of 16 oncoprotein-reactive archived TILs (9 from cervical cancers and 7 from other cancers) displayed HPV-16/HPV-18 cross-reactivity. None of the 10 oncoprotein-reactive TCRs displayed HPV-16/HPV-18 cross-reactivity.

**Conclusions and Relevance:**

Cervical cancers that tested positive for HPV-18 were common in this study and may be common in other US clinical trial populations. Results showed that HPV-16/HPV-18 intergenotype T-cell cross-reactivity of T cells from HPV-16–positive and HPV-18–positive cancers was uncommon. These findings support clinical trial designs in which the HPV type targeted by a therapeutic vaccine is matched with the HPV type of a cancer and suggest a change is necessary in the design of active clinical trials.

## Key Points

**Question:**

Does the cross-reactivity of T cells against the human papillomavirus (HPV) oncoproteins in HPV-positive cancers support current immunotherapeutic vaccine clinical trial designs in which the HPV type of the tumor is not matched with the HPV type of the vaccine?

**Findings:**

In an analysis of 16 samples, HPV-reactive tumor-infiltrating lymphocytes from patients with HPV-positive cancers displayed HPV-16/HPV-18 oncoprotein cross-reactivity in 1 of 16 samples. None of the 10 HPV-reactive T-cell receptors from the researchers’ library exhibited HPV-16/HPV-18 oncoprotein cross-reactivity.

**Meaning:**

The low frequency of HPV-16/HPV-18 T-cell cross-reactivity supports clinical trial designs that match the HPV type of therapeutic vaccines to that of patient tumors.

## Teaser

This study characterizes the prevalence of human papillomavirus 16 (HPV-16) and HPV-18 metastatic cervical cancers among National Cancer Institute (NCI) trial participants and the cross-reactivity of archived T cells between the 2 HPV types to determine whether treating patients with an HPV-16–targeted vaccine would be effective for women with HPV-18 cancers.

## Introduction

Cervical cancer is a difficult-to-treat malignant neoplasm that causes the deaths of more than 4000 women in the United States each year.^1^ It is caused by infection with high-risk human papillomavirus (HPV), which transforms epithelial cells through the actions of the HPV E6 and E7 oncoproteins.^2^ These oncoproteins are constitutively expressed by cervical cancer cells and recognized as immunologically foreign by human T cells; therefore, they are attractive targets for immunotherapy.^3^ The most-studied immunotherapy for specific targeting of these oncoproteins has been therapeutic cancer vaccines.^4,5,6,7,8,9,10^ This immunotherapeutic approach is intended to stimulate an attack on the tumor by E6- and E7-specific T cells from the patient’s endogenous T-cell repertoire. In some clinical trials, including active multicenter studies,^7^ vaccines that target HPV-16 E6 and/or E7 are undergoing testing in patients with cervical cancer regardless of the HPV type harbored by the patient’s tumor.^4,5,6^

At least 13 HPV types cause cancer, including types 16, 18, 31, 33, 35, 39, 45, 51, 52, 56, 58, 59, and 68.^11^ In the United States, HPV-16 is the most common (56%) and HPV-18 is the second most common (13%) type of cervical cancer.^12^ In other common HPV-associated cancers, such as head and neck and anal cancers, HPV-16 is overwhelmingly (>80%) the dominant oncogenic type.^13,14^ The viruses are members of different species (HPV-16 from species 9 and HPV-18 from species 7), and their oncoprotein sequences are nonidentical.^11^ For patients with HPV-18–positive cancers, a clinical trial strategy of vaccinating all patients with an HPV-16 oncoprotein–targeted vaccine therefore relies on cross-reactivity against nonidentical T-cell epitopes. A certain level of cross-reactivity is inherent to the T-cell arm of adaptive immunity because T-cell receptors (TCRs) can exhibit promiscuity in peptide recognition.^15^ However, whether robust intergenotype HPV-16/HPV-18 oncoprotein T-cell cross-reactivity exists in patients with metastatic cervical cancer is not well studied.

We sought to examine the immunological foundation for the strategy of treating all comers with metastatic cervical cancer with an HPV-16–targeted vaccine. Because research on the prevalence of high-risk HPV genotypes in HPV-associated cancers has mainly been based on the study of primary rather than metastatic tumors, we first determined the frequency of HPV-16–positive and HPV-18–positive tumors among patients with metastatic cervical cancer referred for immunotherapy clinical trials at our cancer center. Next, we evaluated whether a panel of archived HPV oncoprotein–specific tumor-infiltrating lymphocytes (TILs) expanded from HPV-16–positive or HPV-18–positive tumors possessed HPV-16/HPV-18 oncoprotein cross-reactivity and could therefore potentially be stimulated to attack tumors caused by both HPV types. Finally, to assess the potential cross-reactivity of individual TCRs, rather than mixed T-cell populations, we tested the HPV-16/HPV-18 cross-reactivity of an archived panel of HPV-16 and HPV-18 oncoprotein–specific TCRs.

## Methods

### Patient Samples

The 65 patients included in the HPV type–prevalence analysis were screened for clinical trials according to National Cancer Institute (NCI) protocol 99-C-0128 at the NCI in Bethesda, Maryland, between April 24, 2012, and March 18, 2015. The 16 samples used to generate archived TILs were separately procured according to NCI protocol 03-C-0277 between May 9, 2012, and September 2, 2014, with approval of the Office of Human Subjects Research Determination 11503. All protocols were approved by the NCI institutional review board, and written consent was obtained from all participants. Race and/or ethnicity was self-reported from predetermined options. Patient leukapheresis and tumor samples were collected and processed by the National Institutes of Health Surgery Branch TIL laboratory. Archived TILs used in this study were grown from fresh tumor samples and selected as previously described.^16^ The 16 samples tested for cross-reactivity represented our complete bank of TILs with known HPV oncoprotein reactivity and sufficient material for assays. Of the 16 TILs tested, 10 TILs were generated from HPV-16–positive tumors (3 from cervical cancers and 7 from other cancers) and 6 TILs were generated from HPV-18–positive tumors (all from cervical cancers).

### HPV Typing

The HPV genotype of the 65 patients screened was determined by a review of medical records and/or by testing of formalin-fixed, paraffin-embedded tumor samples at the Massachusetts General Hospital Department of Pathology using the cobas 4800 System (Roche).

### TCR Transduction

We isolated TCR sequences from archived TILs or cervix-infiltrating lymphocytes by methods previously described.^17,18^ From our library of TCRs, only those with strong HPV oncoprotein reactivity were included in this analysis (eTable in the Supplement). Genes encoding TCRs modified to include murine constant regions were synthesized and cloned into the MSGV1 retroviral vector (GeneOracle). Retroviruses encoding each TCR were used to transduce autologous or healthy donor peripheral blood mononuclear cells (PBMCs) as previously described.^18^ Briefly, MSGV1-TCR vectors and RD114 envelope plasmid were used to generate transient retroviral supernatant from 293GP cells. The PBMCs were stimulated for 2 days using 50 ng/mL soluble CD3 antibody (clone OKT3; Miltenyi Biotec) and 300 IU/mL interleukin 2. Stimulated cells were transduced using plates coated with RetroNectin (Clontech). Transduction was verified by flow cytometry using an anti–mouse constant region beta antibody (eBioscience). Transduced T cells were used in immunological assays 6 to 10 days after transduction.

### Generation of Target Cells for Immunological Assays

Dendritic cells (DCs) were generated from CD14^+^ cells isolated from PBMCs with CD14 MicroBeads (Miltenyi Biotec) and subsequent culturing with 1000 IU/mL recombinant human granulocyte-macrophage colony-stimulating factor (Peprotech) and 500 IU/mL human recombinant interleukin 4 (Peprotech), as described previously.^17,18^ Epstein-Barr virus lymphoblastoid cell lines (EBV-LCLs) were generated from patient PBMCs using the viral supernatant from the B95-8 producer cell line (ATCC) using standard procedures and cultured in Iscove’s Modified Dulbecco’s Medium (Life Technologies) supplemented with 10% fetal calf serum (HyClone) and standard additives.

### Immunological Assays

To test for reactivity of TILs and TCR-transduced T cells against the HPV-16 and HPV-18 E6 and E7 antigens, DCs or EBV-LCLs were pulsed overnight with pools of 15-mer peptides overlapping by 11 amino acids spanning the entire proteins (Miltenyi Biotec), or electroporated with in vitro transcribed RNA (Invitrogen) generated per the manufacturer’s protocol from the full-length HPV oncoprotein genes. For TIL analysis, autologous DCs were used exclusively as targets. For TCR analysis, EBV-LCLs generated from the patient from whom the TCR was isolated were used when possible; otherwise, human leukocyte antigen–matched EBV-LCLs for that particular TCR were used (eTable in the Supplement). The TILs or TCR-transduced T cells (range, 1000-50 000 cells) were cocultured with the pulsed or electroporated DCs or EBV-LCLs (range, 30 000-60 000 cells) for 20 to 24 hours. Interferon-γ was measured by enzyme-linked immunosorbent assay (Thermo Fisher Scientific) or enzyme-linked immunospot assay (Mabtech) per the manufacturers’ instructions.

### Statistical Methods

Data significance was assessed using a 1-way analysis of variance analyzed in GraphPad Prism 7 software. The threshold for statistical significance was set at *P* < .05.

## Results

The median (range) age of 65 referred patients with cervical cancer was 44 (24-64) years. Ethnicity was self-reported for 39 of 65 patients; 35 (89.7%) were white, 3 (7.7%) were Asian, and 1 (2.6%) was American Indian/Alaskan Native. Histologic tumor subtype was recorded for 41 of 65 patients; 25 (61.0%) were squamous cell carcinomas, 12 (29.3%) were adenocarcinomas, 2 (4.9%) were adenosquamous cell carcinomas, and 2 (4.9%) were neuroendocrine tumors. Assessment of HPV genotype revealed that 39 of 65 patients (60.0%) had metastatic cervical cancers that harbored HPV-16 and 21 patients (32.3%) had cancers that harbored HPV-18. Thus, a substantial proportion of patients with cervical cancer had HPV-18–positive cancers, and for these patients it would be important to know whether an HPV-16–targeted vaccine might stimulate HPV-18–reactive T cells.

**Figure 1.**
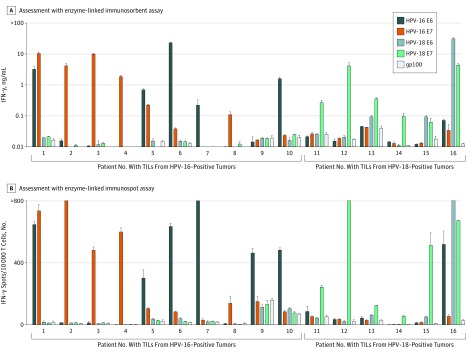
Screening of Tumor-Infiltrating Lymphocytes (TILs) for Cross-reactivity Between Human Papillomavirus 16 (HPV-16) and HPV-18 Oncoproteins Tumor-infiltrating lymphocytes grown from metastatic tumors of patients with HPV-16–positive (n = 10) or HPV-18–positive (n = 6) cancers were cocultured overnight with autologous dendritic cells pulsed with peptide pools (15-mer peptides overlapping by 11 amino acids) spanning the full length of the indicated antigen. Patients 1 through 3 and 11 through 16 had metastatic cervical cancer, while patients 4 through 10 had other types of metastatic cancer. Melanocyte protein PMEL (also known as gp100) was used as a negative control. Reactivity was assessed by interferon-γ (IFN-γ) enzyme-linked immunosorbent assay (A) and IFN-γ enzyme-linked immunospot assay (B). Error bars represent standard deviations of duplicate wells.

To investigate the theoretical potential for oncoprotein vaccines to induce HPV-16/HPV-18 cross-reactive T-cell responses, we studied archived HPV oncoprotein-reactive TILs from the tumors of 16 patients with HPV-16–positive or HPV-18–positive metastatic cancers.^16^ In initial assessment, TILs from 0 of 10 HPV-16–positive tumors (including 3 cervical cancers and 7 other cancers) demonstrated specific HPV-18 E6 and/or E7 reactivity, and TILs from 0 of 6 HPV-18–positive tumors (all cervical cancers) demonstrated specific HPV-16 E6 and/or E7 reactivity (Figure 1A). Further investigation aimed to detect low frequencies of antigen-reactive T cells confirmed that TILs from 0 of the 10 HPV-16–positive tumors demonstrated HPV-18 E6 and/or E7 reactivity. However, TILs from 1 of the 6 HPV-18–positive tumors demonstrated low-frequency HPV-16 E6 reactivity (Figure 1B). Examination of HPV-specific T-cell clones isolated from these HPV-18 E6 and HPV-16 E6 reactive T-cell populations revealed a single CD4^+^ T-cell clonotype that targeted both HPV-18 and HPV-16 E6 (eFigure in the Supplement). In summary, the finding of HPV-16/HPV-18 oncoprotein cross-reactivity in only 1 of 16 samples suggests that HPV-16/HPV-18 oncoprotein cross-reactivity is uncommon in the TILs of patients with metastatic cancer.

**Figure 2.**
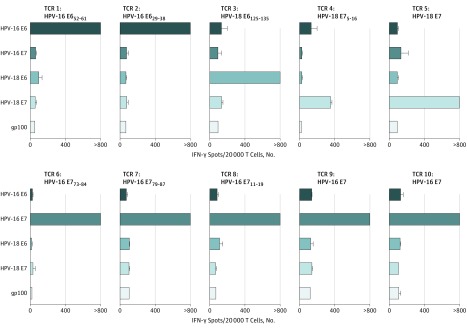
Screening of T-Cell Receptors (TCRs) Targeting the Human Papillomavirus (HPV) Oncoproteins for Cross-reactivity Peripheral blood T cells were retrovirally transduced with TCRs isolated from tumor-infiltrating lymphocytes of HPV-16–positive tumors (n = 7) and HPV-18–positive tumors (n = 3). Transduced T cells were cocultured overnight with autologous or human leukocyte antigen restriction element–matched Epstein-Barr virus lymphoblastoid cell lines (eTable in the Supplement) pulsed with overlapping peptide pools (15-mer peptides overlapping by 11 amino acids) spanning the full length of the indicated antigens. Melanocyte protein PMEL (also known as gp100) was used as a negative control. Reactivity was assessed by interferon-γ (IFN-γ) enzyme-linked immunospot assay. The target epitope of each TCR, when known, is indicated above each graph (eTable in the Supplement). Data are representative of 2 independent experiments. Error bars represent standard deviations of duplicate wells.

Because TILs are composed of a polyclonal population of T cells and thus TCRs with potentially diverse HPV reactivity, we next evaluated the potential HPV-16/HPV-18 oncoprotein cross-reactivity of single-epitope targeted HPV-specific T cells using a panel of HPV-oncoprotein specific TCRs (eTable in the Supplement). None of 10 HPV-specific TCRs evaluated demonstrated HPV-16/HPV-18 oncoprotein cross-reactivity (Figure 2), suggesting that individual TCRs may not be frequently cross-reactive against other HPV oncoproteins.

## Discussion

Various clinical trial designs have been used in the study of HPV oncoprotein vaccines. One strategy is to vaccinate with both the HPV-16 and HPV-18 oncoproteins and thereby target the 2 most common types of high-risk HPV.^19^ Another strategy is to genotype each patient’s tumor and give an HPV-16–directed vaccine only if the tumor is HPV-16 positive.^20^ A third approach is to administer an HPV-16–directed vaccine regardless of the HPV type of the cancer.^4,5,6,7^ For this approach to successfully treat patients with HPV-18–positive cancers, robust HPV-16/HPV-18 T-cell cross-reactivity must be present. Our findings that HPV-16/HPV-18 oncoprotein cross-reactivity occurred in only 1 of 16 TILs from HPV-positive cancers and in 0 of 10 HPV oncoprotein–specific TCRs suggest that vaccination with HPV-16 oncoproteins is unlikely to elicit the strong T-cell responses against the HPV-18 oncoproteins that would be necessary for successful treatment. These data therefore support a strategy either of vaccinating with both HPV-16 and HPV-18 oncoproteins or of HPV genotype matching the patient’s tumor and administered oncoprotein vaccine.

The results of this study are consistent with studies of healthy participants and of vaccinated patients, which have shown low T-cell cross-reactivity between different HPV types.^21,22,23^ Only study of T-cell reactivity in patients with HPV-18–positive tumors treated with HPV-16 oncoprotein vaccines could definitively assess the potential for HPV-16 oncoprotein vaccines to induce T-cell responses against HPV-18 oncoproteins. Nonetheless, the available evidence, including the findings presented here, suggests that vaccination of patients with HPV-18–positive tumors with an HPV-16 vaccine may confer risk with small chance of benefit.^7,24^ Genotype matching of HPV oncoprotein vaccines with patient tumors may optimize the clinical benefit of the therapeutic vaccines and their chance for successful development.

### Limitations

The immunological assays in the study are limited by sample size, with 16 TILs and 10 TCRs evaluated. It is also noteworthy that our data do not rule out T-cell cross-reactivity against every possible complex of an HPV-16/HPV-18 oncoprotein peptide with a human leukocyte antigen molecule. The TCR studies are particularly limited as they are intended to assess cross-reactivity in the context of a single human leukocyte antigen molecule. Nonetheless, the marked paucity of HPV-16/HPV-18 cross-reactive T cells strongly suggests that cross-reactivity is uncommon and that vaccine HPV type should be matched to tumor HPV type.

## Conclusions

Human papillomavirus 18 appears to be common among patients with metastatic cervical cancer seeking enrollment in immunotherapy clinical trials. Cross-reactivity against HPV-16/HPV-18 oncoproteins is uncommon in T cells that infiltrate metastatic HPV-16–positive and HPV-18–positive cancers and in T cells that target the HPV oncoproteins. These data support a change in immunotherapy clinical trial practice to match the HPV type of the oncoprotein vaccine with that of the tumor. These findings have implications for the design of active, multicenter, cooperative group clinical trials.

## Notes

Supplement. **eTable.** Specificities of Tested T-cell Receptors (TCRs) Targeting HPV-16 or HPV-18 Oncoproteins **eFigure.** Cross-reactivity of CD4+ T-cell Clones Isolated From an HPV-18–Positive Tumor **eReferences**
