# Supplementary material for: Human Papillomavirus T-Cell Cross-reactivity in Cervical Cancer: Implications for Immunotherapy Clinical Trial Design
Source: JAMA Netw Open. 2018 Jul 6;1(3):e180706. doi: 10.1001/jamanetworkopen.2018.0706 (PMC6324313; doi:10.1001/jamanetworkopen.2018.0706)
Supplement: Supplement. — eTable. Specificities of Tested T-cell Receptors (TCRs) Targeting HPV-16 or HPV-18 Oncoproteins eFigure. Cross-reactivity of CD4+ T-cell Clones Isolated From an HPV-18–Positive Tumor eReferences [file jamanetwopen-1-e180706-s001.pdf]

Supplementary Online Content

Helman SR, Stevanović S, Campbell TE, et al. Human papillomavirus T-cell cross-reactivity in cervical cancer: implications for immunotherapy clinical trial design. *JAMA Netw Open*. 2018;1(3):e180706. doi:10.1001/jamanetworkopen.2018.0706

eTable. Specificities of Tested T-cell Receptors (TCRs) Targeting HPV-16 or HPV-18 Oncoproteins

eFigure. Cross-reactivity of CD4+ T-cell Clones Isolated From an HPV-18–Positive Tumor

eReferences

This supplementary material has been provided by the authors to give readers additional information about their work.

© 2018 Helman SR et al. *JAMA Network Open.*

eTable: Specificities of tested T-cell receptors (TCRs) targeting HPV16 or HPV18 oncoproteins

TCR*	HLA-Restriction	Target	Epitope	Epitope in Other HPV Type**	HLA-Type target cells used for testing TCR transduced T cells***
**1**	B*35:01	HPV16 E6$_{52-61}$	FAFRDLCIVY	FAFKDLFVVY	A*24:02, 32:01; B*35:01, 40:01; C*03:04, 04:01, DRB1*04:02, 04:04, DQB1*03:02; DPB1*04:01
**2**	A*02:01	HPV16 E6$_{29-38}$	TIHDIILECV	SLQDIEITCV	A*02:01, 29:02; B*15, 45; C*03, 06, DRB1*04:0, 07:01, DQB1*02:02, 03:02; DPB1*04:01, 17:01
**3**	DRB1*15	HPV18 E6$_{125-135}$	RRFHNIAGHYR	QRFHNIRGRWT	A*01, 68; B*07, 08; C*07, 07, DRB1*03:01, 15:01, DQB1*02:01, 06:02; DPB1*04:01, 09:01
**4**	Class II	HPV18 E7$_{5-16}$	KATLQDIVLHLE	TPTLHEYMLDLQ	A*01:01, 25:01; B*08:01, 18:01; C*07:01, 12:03, DRB1*03:01, 16:01, DQB1*02:01, 05:02; DPB1*02:01; 04:01
**5**	Unknown	HPV18 E7	Unknown	Unknown	A*01:01, 02:01; B*08, 44; C*05, 07, DRB1*03:01, 11:01, DQB1*02:01, 03:01; DPB1*01:01, 04:01
**6**	Class II	HPV16 E7$_{73-84}$	HVDIRTLEDLLM	ADDLRAFQQLFL	A*24:02, 32:01; B*35:01, 40:01; C*03:04, 04:01, DRB1*04:02, 04:04, DQB1*03:02; DPB1*04:01
**7**	B*40:01	HPV16 E7$_{79-87}$	LEDLLMGTL	FQQLFLNTL	A*24:02, 32:01; B*35:01, 40:01; C*03:04, 04:01, DRB1*04:02, 04:04, DQB1*03:02; DPB1*04:01
**8**	A*02:01	HPV16 E7$_{11-19}$	YMLDLQPET	IVLHLEPQN	A*02:01, 29:02; B*15, 45; C*03, 06, DRB1*04:0, 07:01, DQB1*02:02, 03:02; DPB1*04:01, 17:01
**9**	B*40	HPV16 E7	Unknown	Unknown	A*03, 31; B*40; C*03, DRB1*01:01, 04:04, DQB1*03:02, 05:01; DPB1*02:01, 04:01
**10**	B*40	HPV16 E7	Unknown	Unknown	A*03, 31; B*40; C*03, DRB1*01:01, 04:04, DQB1*03:02, 05:01; DPB1*02:01, 04:01

*TCR references: TCRs 1, 4, 6, 7 (Ref. 1); TCR 2 (Ref. 2); TCR 8 (Ref. 3)

**HPV16 and 18 oncoprotein amino acid sequences were aligned for epitope comparison using Vector NTI AlignX software (gap opening penalty=10, gap extension penalty=0.1); underline indicates amino acid deviation from the TCR target epitope.

***Target cells used were autologous EBV-LCL for all TCRs, with the exception of TCR8 for which allogeneic HLA-matched target cells were used. TCRs were introduced into autologous peripheral blood T cells for TCRs 1, 4, 6 and 7, while allogeneic peripheral blood T cells were used for TCRs 2, 3, 5, 8, 9 and 10.

Unknown means the HLA-restriction element and/or the targeted epitope were not experimentally defined.

© 2018 Helman SR et al. *JAMA Network Open.*

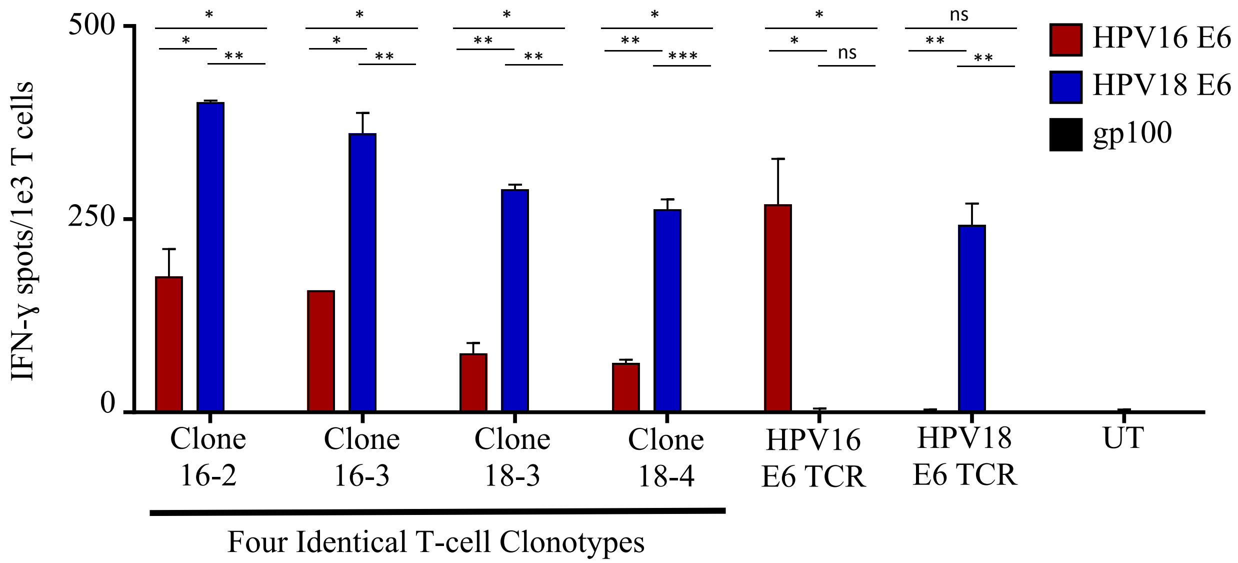

eFigure: **Cross-reactivity of CD4+ T-cell clones isolated from a HPV18+ tumor.** Four identical CD4+ T-cell clones (16-2, 16-3, 18-3, 18-4) isolated from the TIL of patient 16 (HPV18+ tumor) were cocultured overnight with HLA restriction element-matched dendritic cells electroporated with *in vitro* transcribed RNA of the full-length gene of the indicated antigens. Third party peripheral blood T cells retrovirally transduced with a HPV16 E6 specific TCR (HPV16 E6 TCR) or a HPV18 E6 specific TCR (HPV18 E6 TCR) were used as positive controls for recognition of electroporated RNA constructs. Untransduced T cells (UT) and gp100 RNA were used as negative controls. Reactivity was assessed by IFN-ɣ ELISPOT assay. Error bars represent standard deviations of duplicate wells. Significance was assessed by one-way ANOVA. *<0.05, **<0.01, ***<0.001

© 2018 Helman SR et al. *JAMA Network Open.*

eReferences:

1. Stevanović S, Pasetto A, Helman SR, et al. Landscape of immunogenic tumor antigens in successful immunotherapy of virally induced epithelial cancer. *Science*. 2017;356(6334):200-205. doi:10.1126/science.aak9510.
2. Draper LM, Kwong MLM, Gros A, et al. Targeting of HPV-16+ Epithelial Cancer Cells by TCR Gene Engineered T Cells Directed against E6. *Clin Cancer Res*. 2015;21(19):4431-4439. doi:10.1158/1078-0432.CCR-14-3341.
3. Jin B, Campbell T, Draper LM, et al. T cell receptor gene engineered T cells targeting human papillomavirus (HPV)-16 E7 induce regression of HPV-16+ human tumors in a murine model. Poster presented at: 31st Annual Meeting and Associated Programs of the Society for Immunotherapy of Cancer (SITC 2016); November 9-13, 2016; National Harbor, MD.

© 2018 Helman SR et al. *JAMA Network Open.*
